# Robotic surgery in the management of complex pelvic endometriosis

**DOI:** 10.1590/S1677-5538.IBJU.2017.0718

**Published:** 2019-04-01

**Authors:** Guillermo Velilla, Roberto Ballestero, Marcos Gómez, Sergio Zubillaga, Ernesto Herrero, Elena Yllera, José Luis Gutiérrez

**Affiliations:** 1 Department of Urology, Hospital Universitario Marqués de Valdecilla, Santander, Spain;; 2 Department of General Surgery, Hospital Universitario Marqués de Valdecilla, Santander, Spain;; 3 Department of Radiology, Hospital Universitario Marqués de Valdecilla, Santander, Spain

## Abstract

**Introduction:**

Endometriosis consists in the proliferation of endometrial tissue outside of the uterine cavity, predominantly in the ovaries but also in the urinary bladder or bowel. About 10% of fertile women are affected and the main symptoms are pain, menstrual disorders and infertility. Surgery is the treatment option for those symptomatic patients in which medical treatment had no success.

**Material and Methods:**

We report on a case of a 43 - years - old patient without urologic personal history submitted to our office because of a grade - III right - hydronephrosis. The patient, with an endometriosis diagnosis since years, presents chronic pelvic pain with the daily necessity of strong opioids intake. CT scan revealed several endometriosis implants in the uterine wall and rectum that caused right ureteral entrapment. Renography revealed a 24% function in the right kidney. After right nephrostomy a multidisciplinary committee decided surgical intervention. With robotic approach, we performed an hysterectomy with right salpingo - oophorectomy; release, resection and right ureteral reimplantation; anterior resection of the rectum and protective ileostomy. Vaginal extraction of the specimen. In this video we show the key steps of the procedure.

**Results:**

Total operative time: 330 minutes. Total bleeding: 250 cc. Nephrostomy removal: 4 th day. Urethral catheter removal: 5 th day. Patient was discharged in the 7 th day. Ureteral JJ - stent removal: 30 th day. CT urography reveals a permeable ureteral tract with no urine leakage. Renography shows a progressive improvement of the kidney function.

**Conclusions:**

Robotic surgery allows a correct handling of endometriosis, mainly in complex cases. It is a safe and reproducible technique with correct outcomes in selected patients. A multidisciplinary team is required.

## ARTICLE INFO

**Available at: http://www.intbrazjurol.com.br/video-section/20170718_Velilla_et_al**

**Int Braz J Urol. 2019; 45 (video #8): 411-411**

